# *Streptococcus canis* genomic epidemiology reveals the potential for zoonotic transfer

**DOI:** 10.1099/mgen.0.000974

**Published:** 2023-03-31

**Authors:** Davide Pagnossin, William Weir, Andrew Smith, Manuel Fuentes, Juliana Coelho, Katarina Oravcova

**Affiliations:** ^1^​ School of Biodiversity, One Health and Veterinary Medicine, University of Glasgow, Glasgow, UK; ^2^​ Bacterial Respiratory Infection Service, Scottish Microbiology Reference Laboratory, Glasgow Royal Infirmary, Glasgow, UK; ^3^​ Dental Hospital & School, University of Glasgow, Glasgow, UK; ^4^​ Staphylococcus and Streptococcus Reference Section, AMRHAI, Reference Services Division, UK Health Security Agency, London, UK

**Keywords:** *Streptococcus canis*, genomic epidemiology, antimicrobial resistance, virulence, genotyping, zoonosis

## Abstract

*
Streptococcus canis *, a multi-host pathogen commonly isolated from dogs and cats, has been occasionally reported in severe cases of human infection. To address the gap in knowledge on its virulence and host tropism, we investigated *S. canis* genomic epidemiology and report the results of this analysis for the first time. We analysed 59 *S *. *canis* whole genome sequences originating from a variety of host species, comprising 39 newly sequenced isolates from UK sources, along with all (*n*=20) publicly available genomes. Antimicrobial resistance (AMR) phenotype was determined for all 39 available isolates. Genomes were screened for determinants of resistance and virulence. We created a core SNP phylogeny and compared strain clustering to multi-locus sequence typing (MLST) and *S. canis* M-like protein (SCM) typing. We investigated the dataset for signals of host adaptation using phylogenetic analysis, accessory genome clustering and pan-genome-wide association study analysis. A total of 23 % (9/39) of isolates exhibited phenotypic resistance to lincosamides, macrolides and/or tetracyclines. This was complemented by the identification of AMR-encoding genes in all genomes: tetracycline (*tetO* 14 %, 8/59; and *tetM* 7 %, 4/59) and lincosamide/macrolide (*ermB*, 7 %, 4/59). AMR was more common in human (36 %, 4/11) compared to companion animal (18 %, 5/28) isolates. We identified 19 virulence gene homologues, 14 of which were present in all strains analysed. In an *S. canis* strain isolated from a dog with otitis externa we identified a homologue of *S. pyogenes* superantigen SMEZ. The MLST and SCM typing schemes were found to be incapable of accurately representing core SNP-based genomic diversity of the *S. canis* population. No evidence of host adaptation was detected, suggesting the potential for inter-species transmission, including zoonotic transfer.

## Data Summary

Raw read data are available at the European Nucleotide Archive (ENA; www.ebi.ac.uk/ena) under BioProject ID PRJEB55124 and at the Sequence Read Archive (SRA; https://www.ncbi.nlm.nih.gov/sra) under BioProject ID PRJNA865727. Metadata for each strain sequenced and analysed, including origin, sequencing technology and accession number, are reported in Table S1 (available with the online version of this article).

**Impact Statement**

*
Streptococcus canis* is a bacterial pathogen that, although mainly isolated from dogs and cats, can cause disease in a wide range of mammalian species, including humans. Reports of severe cases of *S. canis* infection both in human and in veterinary medicine have been published, with clinical manifestations such as endocarditis, necrotizing fasciitis and septicaemia. Relatively little research has been hitherto conducted to characterize this pathogen due to the low incidence of disease in humans. However, the ever-increased popularity of dogs and cats as family members and the advances in medical science, which are extending the life expectancy of immunocompromised and elderly individuals, may increase human exposure to *S. canis* infection. This study was designed to fill some key knowledge gaps regarding the epidemiology of *S. canis* in humans and companion animals. For the first time, we used whole genome sequence (WGS) analysis to describe the prevalence of antimicrobial resistance and virulence determinants in *S. canis* isolates. We also conducted a WGS-based population analysis, focusing on validation of previously proposed genotyping systems and the detection of signs of host adaptation. We finally provide recommendations on *S. canis* strain typing and we show with a high level of confidence that *S. canis* strains circulating in animal populations can infect humans too.

## Introduction

*Streptococcus canis* is a Gram positive, β-haemolytic, Lancefield Group G *Streptococcus* [1]. While normally found on the skin and mucosal membranes of dogs and cats not showing clinical signs of infection [2], *S. canis* can occasionally be involved in canine and feline skin and soft tissue infections [2] or, on rare occasions, in invasive and severe forms of disease [3–5]. Although dogs and cats appear to be the main reservoirs of this bacterial species, *S. canis* has been isolated from and implicated in disease in several mammalian host species, including cattle [6, 7] in which it is associated with sub-clinical mastitis, and humans [8]. In humans, *S. canis* infections are uncommon but occasionally result in severe clinical manifestations, such as septicaemia [9–11] and endocarditis [12, 13].

Over the past two decades, reports of life-threatening cases of *S. canis *-associated disease in humans [9, 10, 12, 13], together with the ever-increasing popularity of dogs and cats as family pets, have drawn attention towards this bacterial species. A number of studies have investigated *S. canis *, but compared with other streptococcal species, work on this pathogen has been limited and much remains unclear. For instance, while epidemiological studies have provided information regarding the carriage and incidence of infection in pets and humans, to date they have been limited in scope and number [2, 8, 14–16]. The emergence of antimicrobial resistance (AMR) in *S. canis* isolates has also been documented [8, 14, 17, 18], but its burden is unclear and the underlying biological determinants are not fully known. Several virulence mechanisms have been investigated [19–22] but, similarly, little is known about the carriage of virulence genes in the global *S. canis* population. Two main systems for genotypic strain classification, a multi-locus sequence type (MLST) scheme [17] and one based on the allelic variations of the *S. canis* M-like (SCM) gene [23, 24], have been developed. However, neither of these schemes have been tested against high-discrimination typing techniques such as core genome SNP typing. Finally, a lack of host-specificity of *S. canis* has been previously proposed, based on the identification of the same MLST isolates in different host species [23]. However, traditional MLST is based on the allelic variations of only seven housekeeping genes across the entire genome, making it a relatively low-discrimination approach; caution should be exercised in making inferences about bacterial population structure and evolution on the basis of such sparse genetic markers [25].

The use of high-throughput sequencing data, such as whole genome sequencing (WGS), to study bacterial populations has been increasingly employed in recent years and is now considered a key element in our understanding of pathogen epidemiology and evolution [26]. Many of the knowledge gaps surrounding *S. canis* may be addressed by WGS population analysis, which has never hitherto been applied to the study of this bacterial species.

In this work, for the first time a WGS-based analysis of a collection of *S. canis* strains (*n*=59) from different hosts and geographical locations was performed. The presence of AMR genetic determinants was investigated and compared to phenotypic AMR profiles. The presence and distribution of virulence genes was also evaluated. Both MLST and SCM classification systems were assessed against *S. canis* core genome diversity. Finally, evidence of host-adaptation was sought by observing core and accessory genome clustering and determining whether genomic traits were over-represented among isolates from particular hosts through pan-genome-wide association study (GWAS) analysis.

## Methods

### Isolate collection, WGS and sequence assembly

Thirty-nine isolates of *S. canis* from dogs, cats, humans and a seal collected in the UK between 2002 and 2021 were available for this study. All the isolates originated from individual cases of disease and, to our knowledge, they are not epidemiologically linked with each other. Twenty-eight animal-derived isolates were provided by the University of Glasgow Veterinary Diagnostic Services (VDS, Glasgow, UK), one human isolate by the Glasgow Royal Infirmary (GRI, Glasgow, UK) and ten human isolates by UK Health Security Agency (UKHSA, Colindale, UK). The isolates were acquired during routine laboratory diagnostics and kept by the providers. The isolates were anonymized prior to shipping to the authors’ laboratory. All frozen glycerol-stored isolates were grown overnight on Todd-Hewitt Broth (THB) agar (Thermo Scientific) at 37 °C and submitted to the Scottish Microbiology Reference Laboratories (SMiRL, Glasgow, UK) for WGS. The DNeasy 96 Blood and Tissue Kit (Qiagen) was used to extract genomic DNA according to the manufacturer’s instructions. DNA was purified with the QIAsymphony extraction instrument (Qiagen) and quantified using Qubit dsDNA BR Assay Kit on a Qubit 3 Fluorometer. Paired-end sequencing libraries were prepared with a Nextera XT DNA Library Preparation Kit and Index Kit V2 (Illumina), and paired-end sequencing was carried out using Illumina MiSeq technology. Raw sequencing reads were trimmed using ConDeTri [27] and contigs were assembled using SPAdes v 3.14.0 [28]. The quality of the assemblies was assessed using QUAST v 5.0.2 [29] and assembly statistics are reported in Table S1. Assemblies of fewer than 200 contigs and with a total genome length and GC content (%) within ±3 standard deviations of the mean were considered to be of acceptable quality, and this included all the 39 newly generated genomic sequences.

### Genome dataset

All the publicly available genomes of *S. canis* (*n*=20) from the National Center for Biotechnology Information (NCBI) genomes database and the newly generated genomic sequences were analysed in the current study. The publicly available sequences derived from *S. canis* isolates originated from different geographical locations, namely South Korea, Japan, Europe and the USA. Overall, the dataset studied was composed of whole genome sequences from isolates derived from two cattle, nine cats, 32 dogs, 14 humans, one unspecified animal and one common seal. Additional information, including accession numbers, for each sequence included in this work is provided in Table S1.

### Antimicrobial resistance

The presence of genes associated with AMR was determined using ARIBA v 3.1.0 [30] and the CARD database [31], as of August 2021. Only short sequence reads can be analysed by ARIBA, and hence synthetic paired-end short reads were generated using readSimulator [32] from four published genomes (accession numbers: LR590625.1, 42197_B02, 42912_C01, ASM26830v2). Three of these genomes were generated using long-read sequencing technologies and one did not have publicly accessible sequencing reads. ARIBA was also used to extract sequences of *gyrA*, *gyrB*, *parC* and *parE* genes from each genome, using as reference the corresponding genes from the publicly available strain HL_98_2 (accession number ASM1099386v2). Sequence alignments of *gyrA*, *gyrB*, *parC* and *parE* were then produced using MAFFT v 7 [33] and visually inspected with MEGAX v 10.1.7 [34] to assess the presence of mutations associated with quinolone resistance in *S. canis* [18].

Minimum inhibitory concentrations (MICs) to a panel of antibiotics commonly used to treat Gram-positive infections, namely ampicillin, amoxicillin, clindamycin, ceftriaxone, cefotaxime, doxycycline, erythromycin, levofloxacin, meropenem, moxifloxacin, oxacillin, penicillin G, tetracycline and vancomycin, were determined for the 39 available isolates of *S. canis* using broth microdilutions as per EUCAST guidelines [35]. Bacterial cultures were plated and grown on Columbia blood agar plates (Oxoid) for 48 h at 37 °C. For each isolate, saline solutions (0.85 % NaCl, pH 5.5–6.5) were inoculated with bacterial cells to reach a density of 0.5 McFarland (0.44–0.56). Bacterial suspensions were then added to a solution of Micronaut H-Medium broth (BioConnections). Equal volumes of H-Medium broth were then distributed in a 96-well Micronaut-S PHE Co-GP03 plate. Plates were incubated for 22–24 h at 37 °C. Bacterial growth was then measured by using a Multiskan FC Microplate photometer (Thermo Scientific) and MIC values were interpreted by the Micronaut MCN6 software according to the EUCAST breakpoint values v 12.0 [36]. For isolates with tetracycline-resistant phenotypes and in which AMR-conferring genes could not be detected, the command line version of the nucleotide Basic Local Alignment Search Tool (BLASTn) v 2.9.0 [37] was used to investigate the acquisition of rRNA gene mutations.

### Virulence genes

The presence of genes homologous to known bacterial virulence factors was assessed in this collection of genomes using the command line version of BLASTn, coupled with the virulence factor database (VFDB) [38], as of August 2021. A positive match was considered to be one with at least 20 % sequence identity, at least 90 % gene coverage, a bit score >50 and an e-value <10^−10^ [39]. This parameter choice allowed for a conservative approach that maximized the specificity of the search.

### Strain typing

MLSTs were determined for all the 59 genomes using the PubMLST database [40] and the software mlst [41]. SCM sequences were extracted from all genomes using ARIBA and assigned an SCM type according to the typing scheme developed by Fukushima *et al.* [24]. The *scm* gene sequences of all genomes analysed are provided in Table S2.

### Phylogenetic analysis

A core genome SNP alignment of all the 59 whole genome sequences in the current database was generated with snippy v 4.4.5 [42], using the genome HL_98_2 as a reference (accession number: ASM1099386v2). A maximum-likelihood phylogenetic analysis with 100 standard non-parametric bootstrap replicates was then carried out using IQ-TREE v 2.1.4 [43] to produce a core SNP phylogenetic tree. The tree was visualized and annotated using RStudio v 2022.7.2.576 [44] and the packages ggtree [45] and phytools [46]. An outgroup of four *Streptococcus dysgalactiae equisimilis* genomes (accession numbers: ASM1419289v1, 44503_D02, 42197_A02, 46166_D01) was used to root the tree. Core genome SNP (CGS) types were identified in the core SNP phylogeny using TreeCluster [47] with a threshold of 0.017, 0.009 and 0.004 and the Max method. These settings allowed for the detection of three sets of clusters with, respectively, a maximum of 2000, 1000 and 500 pairwise core SNP differences between isolates. IQ-TREE was then used to generate two additional core SNP phylogenies, one constrained to be monophyletic for the MLSTs and one constrained to be monophyletic for the SCM types.

### Comparative phylogenetic analysis

Both constrained phylogenies were compared to the unconstrained core SNP tree using an Approximately Unbiased (AU) test [48] with 10 000 non-parametric bootstrap replicates on IQ-TREE. The AU test evaluates different tree topologies under the null hypothesis that the trees tested provide an equally good explanation of the dataset in use. The accuracy of strain clustering according to the MLST and SCM schemes was then determined in comparison to the newly identified CGS types in each of the three sets of phylogenetic clusters using the adjusted Wallace (AW) coefficient [49] with 95 % confidence intervals (CIs) on the Comparing Partitions website [50]. Finally, BLASTn was used to characterize the differences in the MLST genes between closely related core SNP genotypes with differing sequence type.

### Pangenome-wide association analysis

Genomes were annotated using Prokka v 1.14.6 [51]. An *S. canis* pangenome was then generated with Panaroo using default settings of the strict mode [52]. Scoary v 1.6.16 [53] was later used to carry out a pan-GWAS analysis to investigate the potential overrepresentation of specific genetic markers in strains from different host species. For the pan-GWAS analysis, *P*-values were adjusted using the Benjamini–Hochberg method [54], to correct for false positives while undertaking multiple statistical testing.

### Accessory genome network

The accessory gene diversity of the population studied was determined with GraPPLE [55], which calculates the pairwise similarity between genomes, expressed as a proportion of shared accessory genes. Accessory genes were considered those shared by ≤99 % of the analysed genomes, based on the pangenome analysis results. The pairwise distance matrix produced by GraPPLE was visualized on Graphia v 2.2 [56] in the form of a network. For proper network visualization, edges were reduced using the k-nearest neighbour (k-NN) algorithm, calculated with edge weight, *k*=5 and descending order.

## Results

### Antimicrobial resistance

We initially focused on the detection and identification of AMR-conferring genes in the genomic sequences. A total of six different genes associated with AMR were found among the genomes analysed (Table 1). These genes were *ermA* and *ermB*, associated with macrolide, lincosamide and streptogramin B (MLSb) resistance [57], *lsaC*, associated with lincosamide resistance [58], and *tetM*, *tetO* and *tetS*, all associated with tetracycline resistance [59]. Seventeen of the 59 genomes (29 %) were positive for the presence of at least one AMR gene. Only three genomes were positive for more than one AMR-conferring gene, all having both the *ermB* and *tetO* genes. The most common AMR gene in the dataset was *tetO* (8/59) and tetracycline resistance appeared to be the most prevalent, based on the genotype (14/59). If the results from publicly available genomes are excluded, which may have specifically been sequenced because of their AMR characteristics, the prevalence of at least one AMR-conferring gene in our cohort of *S. canis* isolates is 23 % (9/39). None of the previously reported single point mutations in the quinolone resistance-determining regions (QRDR) of *gyrA*, *gyrB*, *parC* and *parE* associated with quinolone resistance was detected in this cohort of genomes.

**Table 1.** Genomic determinants of AMR detected among the 59 *S. canis* genomes analysed

AMR determinant	Newly generated sequences	Publicly available sequences	Total
*ermA*	1/39 (3 %)	0/20 (0 %)	1/59 (2 %)
*ermB*	2/39 (5 %)	2/20 (10 %)	4/59 (7 %)
*lsaC*	1/39 (3 %)	0/20 (0 %)	1/59 (2 %)
*tetM*	1/39 (3 %)	3/20 (15 %)	4/59 (7 %)
*tetO*	6/39 (15 %)	2/20 (10 %)	8/59 (14 %)
*tetS*	0/39 (0 %)	2/20 (10 %)	2/59 (3 %)
*ermB +tetO*	2/39 (5 %)	1/20 (5 %)	3/59 (5 %)
Quinolone-conferring mutations	0/39 (0 %)	0/20 (0 %)	0/59 (0 %)
Any AMR determinant	9/39 (23 %)	8/20 (40 %)	17/59 (29 %)

**Fig. 1.**
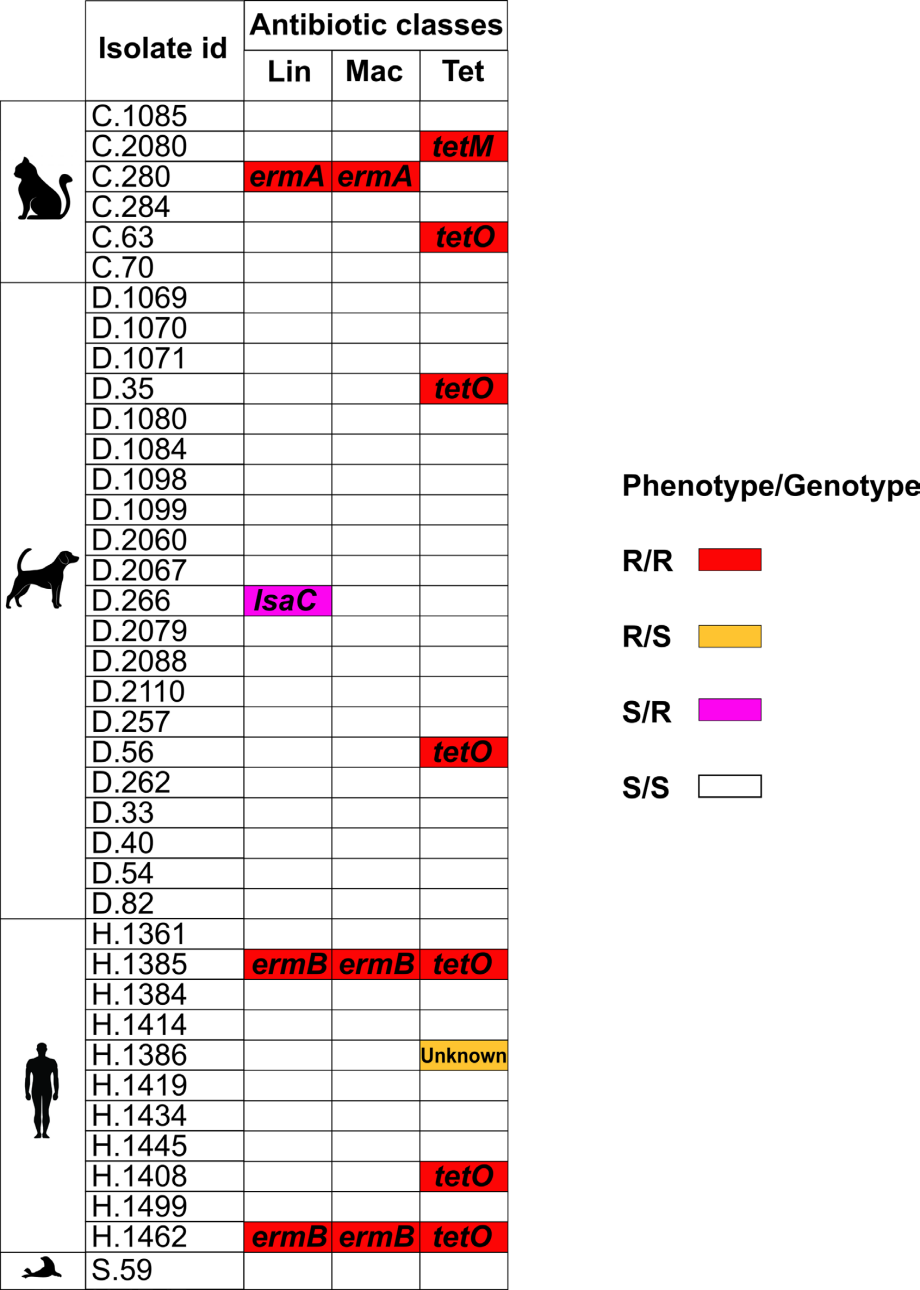
Concordance between AST results (phenotype) and presence of AMR-associated genes (genotypes) in the 39 isolates tested. Each row represents one isolate and isolates are grouped based on the host (cat, dog, human and seal) from which they were collected. Since phenotypic resistance was found only towards lincosamides (clindamycin), macrolides (erythromycin) and tetracyclines (doxycycline and tetracycline), only results referring to these antibiotic classes are reported. Genomic determinants of AMR are reported for isolates showing phenotypic and/or genotypic resistance profiles. Lin, lincosamides; mac, macrolides; tet, tetracyclines; R, resistance; S, sensitivity.

Thirty-nine *S. canis* isolates were tested for antimicrobial sensitivity towards 14 antibiotics representative of six antibiotic classes, namely β-lactams, fluoroquinolones, glycopeptides, lincosamides, macrolides and tetracyclines. MIC values for the antibiotics tested are reported in Table S3. Based on the values reported in the EUCAST breakpoint table v 12.0 for Group G streptococci, resistance was detected against clindamycin (lincosamides), erythromycin (macrolides), tetracycline and doxycycline (tetracyclines). All isolates tested were fully sensitive to β-lactams. The concordance between antimicrobial susceptibility testing (AST) and genomic inference was very high (95 % agreement) and the carriage of AMR-associated genes among the 39 isolates tested is illustrated in Fig. 1. One isolate that was predicted to be resistant to lincosamides based on genotype was found to be fully susceptible to all antibiotics tested. Nine of 39 isolates tested (23 %) were resistant to at least one antibiotic and for eight of these a genomic determinant of AMR could be identified. Isolate H.1386 was found to be resistant to tetracycline and no resistance-conferring gene was detected within its genome. The rRNA sequences within the genome of this isolate were then compared to all the other *S. canis* whole genome sequences, in order to detect or rule out possible tetracycline resistance-conferring ribosomal mutations. Twelve other whole genome sequences carried 5S, 16S and 23S rRNA sequences identical to those of isolate H.1386. Eight of those 12 genomes derived from isolates in our collection, all of which tested as fully susceptible to tetracyclines in the present study, thus excluding ribosomal mutation as causing resistance in this isolate.

### Pangenome analysis and virulence gene identification

The newly generated *S. canis* pangenome comprised 1 432 core genes, defined as those shared by at least 99 % of genomes. The majority of the remaining 2 994 genes that constituted the *S. canis* accessory genome were found to be shared by ten or fewer strains (Fig. S1).

**Fig. 2.**
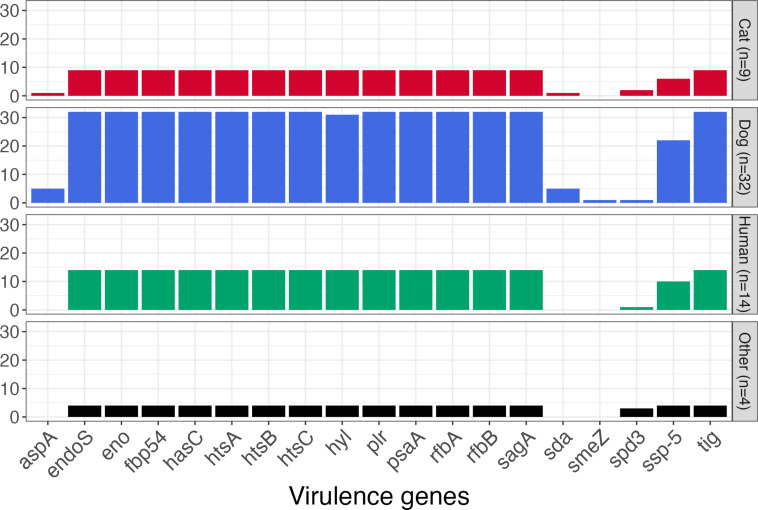
Number of genomes, grouped by host species, carrying genes homologous to known virulence genes within the VFDB. The group ‘Other’ includes two genomes from bovine isolates, one genome from an unspecified animal isolate and one genome from a seal isolate.

A total of 19 genes homologous to known virulence genes within the VFDB was found among the 59 genomes analysed (Fig. 2). Fourteen of these genes (74 %) were detected in every genome. One gene, homologous to the hyaluronidase-encoding gene *hyl*, was detected in all but one genome. Forty-two whole genome sequences were positive for the carriage of a homologue to the *ssp-5* gene, which encodes an agglutinin receptor. Seven genomes carried homologues to one or more of the following: *aspA*, *sda, spd3* and *smeZ*. The *smeZ* gene, whose product is the streptococcal mitogenic exotoxin Z, is found in some *S. pyogenes* strains and when present it is usually integrated into the chromosome. In the current genome dataset, only one isolate was found to carry *smeZ*. Visual inspection of the contig containing the gene indicated that *smeZ* was encoded in a chromosomal locus (Fig. S2). The *sda* gene, conversely, is traditionally considered a phage-associated gene that has been described in different streptococcal species, including *S. pyogenes *. Based on a BLASTn search of the NCBI non-redundant nucleotide database, the closest variant to the *S. canis sda* genes appeared to be *S. pyogenes sdaD2*. Moreover, the presence of phage-like genes in proximity to *sda*, confirmed by both BLASTn searches and Prokka genome annotations, suggested a phage-mediated acquisition of this gene in *S. canis *. Similarly, the *spd3* gene, which is a well-known phage-associated virulence factor of *S. pyogenes *, was found in proximity to phage-like genes in *S. canis *, offering a plausible explanation as to why it was detected in just a limited number of isolates. All the virulence genes found in the current WGS dataset have been described in other pathogenic streptococcal species, such as *S. pyogenes *, *S. agalactiae* and *S. pneumoniae* [38]. The *scm* gene, encoding the universally present virulence factor *S. canis* M-like protein [23], is not currently represented in the VFDB. The presence of such a gene, however, was confirmed in all isolates using as a reference publicly available *scm* sequences and command line BLASTn. A breakdown of all virulence genes detected in each genome is provided in Table S4.

### Population analysis

**Fig. 3.**
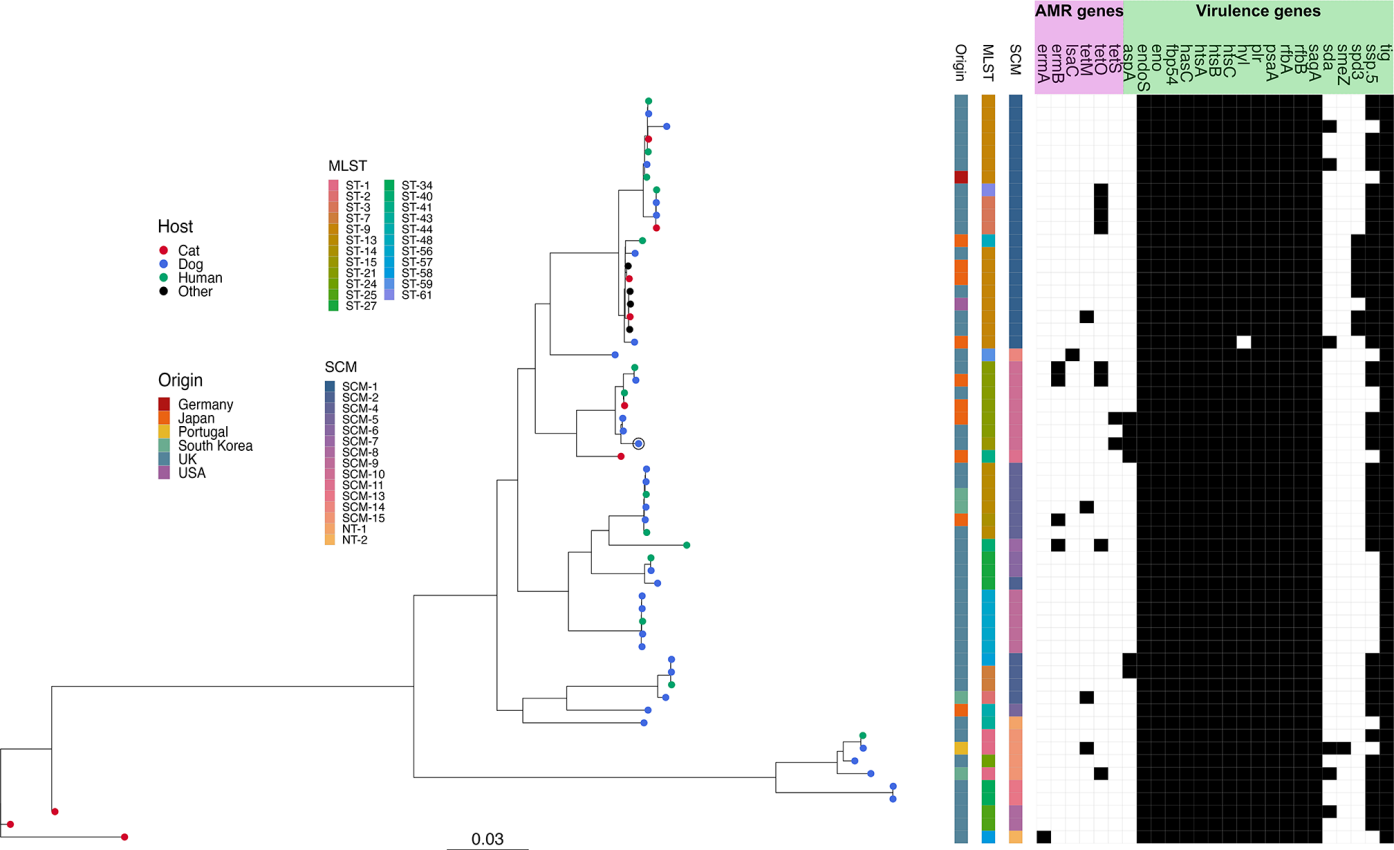
Maximum-likelihood core SNP phylogenetic tree of the 59 whole genome sequences analysed. Isolates are coloured according to the host species from which they were isolated. The reference genome used for phylogenetic analysis, namely D.HL_98_2 (accession number: ASM1099386v2), is highlighted on the tree by a black outer circle. Tree annotations regarding the isolates’ geographical origin and classification according to MLST and SCM Fukushima types are reported. NT stands for new types and indicates those *scm* alleles that were not classified in the work of Fukushima *et al.* A heatmap shows which genomes possess (black squares) or which do not possess (white squares) AMR and virulence genes detected in previous analyses.

A core SNP maximum-likelihood phylogeny was built with the aim of providing a high-resolution representation of the evolutionary relationships among isolates (Fig. 3). Since the two existing typing schemes proposed for *S. canis *, the MLST and SCM systems, have never been validated against a highly accurate typing technique such as core genome SNP typing, both MLST- and SCM-constrained core SNP maximum-likelihood phylogenies were also reconstructed and compared to the unconstrained core SNP tree.

The results of the AU statistical test used to compare the phylogenies indicate that both constrained trees are significantly different from the unconstrained one (Table 2). It can therefore be concluded that the clusters predicted by the MLST and SCM schemes are not a perfect fit to the core SNP data. This result was further confirmed by visual inspection of the annotated core SNP tree (Fig. 3). It was apparent that certain isolates which were genetically closely related to others had been classified as different sequence types (STs), such as the set of ST3, ST48 and ST61 isolates which were found to be nested within a predominantly ST9 cluster. In such instances, the acquisition of a limited number of SNPs was responsible for most discrepancies between core SNP phylogenetic analysis and MLST classification. In one case, however, signs of MLST gene recombination between phylogenetically distant isolates were detected.

**Table 2.** AU statistical comparison of MLST- and SCM-constrained phylogenies using the core SNP unconstrained tree as a reference Log likelihood (ln L), difference in log likelihood and *P*-values are reported for each tree comparison.

Tree tested	ln L	Diff ln L	*P*-value
Core SNP MLST-constrained	−501089.9	2631	6.68×10^−7^
Core SNP SCM-constrained	−508213.29	9754.4	1.48×10^−40^

Eighteen CGS types, highlighted in Fig. S3, were found in the core SNP phylogeny by TreeCluster using a threshold value of 0.017. Twenty-two and 29 CGS types were identified, respectively, using 0.009 and 0.004 as threshold values. The AW coefficient of the MLST and SCM schemes compared to each of the three CGS clustering sets is presented in Table 3. The AW coefficient can be interpreted as the probability of two isolates belonging to the same group according to one scheme (MLST or SCM) and also belonging to the same group according to another scheme (CGS). In each of the three scenarios tested, MLST typing appeared more accurate than SCM classification. The 95 % CI, however, overlapped or nearly overlapped in all scenarios, indicating that there is no strong evidence that MLST typing is significantly more accurate than SCM typing.

**Table 3.** AW coefficient with 95 % CI of SCM and MLST typing compared to each set of CGS types

TreeCluster threshold value	No. of CGS types	Maximum intra-cluster pairwise distance (SNPs)	AW SCM→CGS (95 % CI)	AW MLST→CGS (95 % CI)
0.017	18	2000	0.540 (0.467–0.613)	0.575 (0.495–0.654)
0.009	22	1000	0.346 (0.255–0.437)	0.532 (0.447–0.617)
0.004	29	500	0.244 (0.158–0.330)	0.369 (0.248–0.489)

**Fig. 4.**
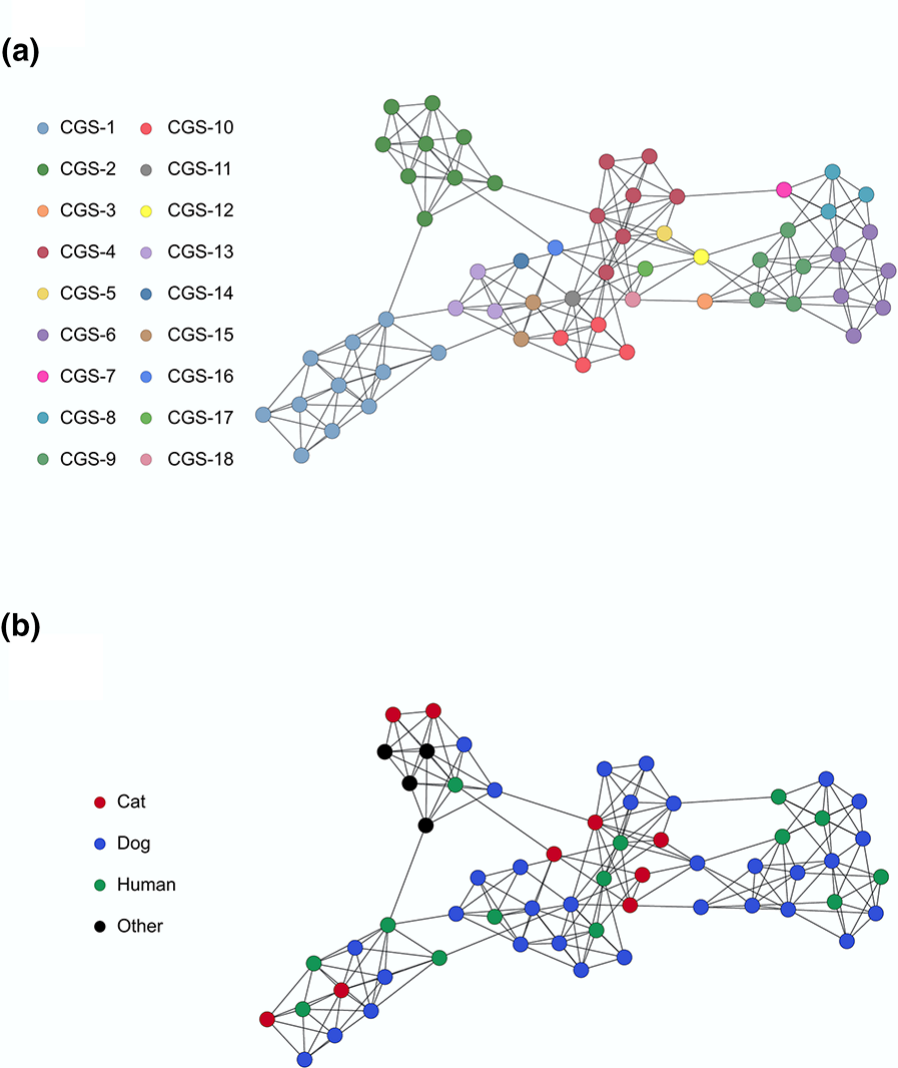
Accessory genome network for the *S. canis* strains investigated. Each dot represents the accessory genome of a bacterial isolate. Dots connected and/or clustering have a similar accessory genome. (a) Dots are coloured according to the CGS clusters identified by TreeCluster with a threshold value of 0.017 (Fig. S3). (b) Dots are coloured according to the isolate host species.

An *S. canis* core SNP phylogeny was also used to investigate the relatedness of isolates from different host species. The core SNP tree indicated that isolates from different hosts frequently cluster together, suggesting a lack of host adaptation in the *S. canis* cohort analysed (Fig. 3). In one case, the core SNP distance between a human and a dog isolate was found to be as low as 25 loci. All pairwise core genome SNP distances in the current genome dataset are shown in Fig. S4. Diversity within the *S. canis* accessory genome was also evaluated. A network based on the presence/absence of accessory genes was created and is depicted in Fig. 4. Overall, the accessory gene content of *S. canis* isolates was not reflective of strain relatedness based on core SNP profile (Fig. 4a), although a degree of structure was evident. In agreement with the core SNP phylogenetic tree, the accessory gene network did not show any signs of host specificity in the current *S. canis* dataset (Fig. 4b). This apparent lack of host specificity was further supported by the results of the pan-GWAS analysis, which revealed no specific genomic traits overrepresented in different host groups across the 59 genomes analysed.

## Discussion

*S. canis* has been recognized for decades as a pathogen of multiple mammalian species, but many aspects of its biology and disease epidemiology remain unclear. In this work, for the first time, a genomic-based approach was used to study *S. canis *, with the aim of clarifying some important epidemiological characteristics such as AMR prevalence, virulence gene distribution and overall population structure. This study represents the largest *S. canis* genome collection (*n*=59) currently available.

Given the limited knowledge regarding AMR prevalence and its genetic determinants in *S. canis *, the available collection of genomes was scanned for the presence of AMR-encoding genes and mutations previously associated with quinolone resistance [18]. Six resistance-encoding genes, *ermA*, *ermB*, *lsaC*, *tetM*, *tetO* and *tetS*, were detected across the genomic dataset. All these genes, except *lsaC*, have previously been detected in *S. canis* isolates resistant to macrolides, lincosamides and tetracyclines [14]. When expressed, the gene *lsaC* confers high levels of lincosamide resistance through an efflux mechanism, as has previously been described in *S. agalactiae* strains [58]. In our study, 17 genomes carried at least one AMR gene and only three genomes carried two. None of the previously described mutations associated with quinolone resistance in *S. canis* isolates was detected in this collection of genomes. Phenotypic testing revealed resistance towards lincosamides, macrolides and tetracyclines, which appears to be commonly encountered in *S. canis* strains isolated from dogs, cats and humans [8, 14, 17]. Of the 39 isolates tested, 23 % (9/39) were resistant to at least one antibiotic class. The prevalence of resistance in the current study was lower than that described in the literature, where, for example, tetracycline resistance is estimated to be expressed by approximately 30–40 % of the isolates [8, 14, 17]. The genomic predictions of AMR generated in the current work matched the phenotypic results for eight out of nine isolates. One canine isolate was fully sensitive to all antibiotics tested, despite lincosamide resistance being anticipated based on carriage of the *lsaC* gene [58]. This may suggest that the *lsaC* gene was not expressed or another compensatory mechanism prevented the phenotypic lincosamide resistance being expressed *in vitro*, although lincosamide resistance *in vivo* cannot be excluded. One human isolate, in contrast, showed tetracycline resistance without carrying any known tetracycline resistance gene. Since the acquisition of rRNA mutations that can be associated with tetracycline resistance was ruled out in our isolate, we suggest that the acquisition of a novel resistance mechanism could be responsible for the observed resistance phenotype [60]. A difference in the prevalence of AMR in human, 36 % (4/11), vs. companion animal isolates, 18 % (5/28), was noted. Although this result may be biased by the underlying reasons for which samples were collected, by the year, host, country of isolation and small sample size, it may be hypothesized that antimicrobial use in human medicine has increased the selective pressure on *S. canis* strains carried by humans. Importantly, no β-lactam resistance was encountered in this study, suggesting that first-line antimicrobials such as amoxicillin clavulanate and penicillin G are still a suitable option to treat *S. canis* infections.

In the present study, the chosen thresholds to identify virulence genes using BLASTn and VFDB were much stricter than those used in a previous work on a single *S. canis* genome [61] and, as a result, the number of positive matches we obtained was considerably lower than those observed in the previous work. Nineteen virulence gene homologues were found in the present *S. canis* genome dataset. In other pathogenic streptococci, the corresponding virulence genes are involved in tissue adhesion, tissue invasion and immune response evasion [62, 63]. Seventeen of the gene homologues detected (i.e. all except *aspA* and *ssp-5*) correspond to virulence genes also found in *S. pyogenes* [63], and nine of these genes (*eno*, *fbp54*, *hasC*, *hyl*, *plr*, *rfbA*, *rfbB*, *sagA* and *ropA*) are considered part of the *S. pyogenes* core genome [64], providing further evidence of the close evolutionary relatedness between *S. canis* and *S. pyogenes* [65]. A gene homologous to an *S. pyogenes* superantigen, *smeZ*, was found in an *S. canis* genome (accession number: SAMEA4968065). Streptococcal superantigens are potent exotoxins that play an important role in severe forms of infection [66]. Some superantigens are phage-encoded, allowing for intra- and inter-species recombination events via lateral transduction, but *smeZ* is chromosomally encoded in *S. pyogenes* [67] and, based on our findings (Fig. S2), this is also the case in *S. canis *. The mechanism of acquisition of this gene in *S. canis* is thus unknown. To our knowledge, this is only the second time that a superantigen homologue has been found integrated in an *S. canis* genome [68]. In the current study, only the carriage of homologues to known virulence genes was considered, but the presence of unknown *S. canis *-specific virulence genes or genes that would be identified with less stringent settings cannot be ruled out.

A genome-based population analysis of *S. canis* was then carried out. First, a core-SNP phylogenetic tree was reconstructed, which was used as a reference to validate the MLST and SCM typing schemes. Our findings suggest that both systems fail to represent with high accuracy *S. canis* population diversity. Since both schemes are based on very limited fractions of the bacterial genome, this result is unsurprising and confirms published data [25]. In the present work, we did not find convincing evidence that one scheme is significantly more accurate than the other. In light of our findings, we recommend the use of a core-SNP phylogeny over MLST and SCM systems for high-resolution applications such as outbreak investigation, and we suggest the creation and curation of an SCM database to facilitate basic classification and the identification of *S. canis* lineages.

The core SNP phylogenetic tree was also used to investigate evolutionary relationships among the isolates. No host- or country-specific clustering was observed in the phylogeny, suggesting that the same strains circulate across host and geographical boundaries. In a previous study, strains of *S. canis* sharing the same STs were isolated from multiple species, leading the authors to conclude that this pathogen may circulate among different hosts [17]. The present work supports those findings with a higher degree of confidence and, additionally, takes into account the potential impact of the accessory genome in the process of host adaptation [69]. The accessory genome network produced provides further evidence for an absence of host-specificity among *S. canis* strains. The accessory genome network also revealed that isolates with distant core-genome SNP profiles can share a similar accessory gene content, and vice versa. This finding, together with the considerable size of the accessory genome predicted by Panaroo (Fig. S1), highlights the plasticity of the *S. canis* genome.

Finally, an *S. canis* pangenome was constructed which encompassed 4 426 genes, 1 432 of which classified as the core genome. Compared to the core genome of *S. pyogenes *, which was defined by 1 306 coding sequences from 2 083 isolates in a work by Davies *et al.* [64], the *S. canis* core genome is somewhat larger. This may be related to the limited number of sequences included in the current study (*n*=59). The *S. canis* pangenome was used to perform a pan-GWAS analysis that searched for the over-representation of genetic markers among the different host species. In *S. agalactiae *, for example, host adaptation seems to be driven by the presence of a limited number of genes in specific lineages [70]. In the current work, no genomic traits were significantly associated with specific hosts, although this analysis was based on a limited number of genomes. Nevertheless, our findings regarding core and accessory genome diversity and pan-GWAS analysis indicate a paucity of host adaptation for the pathogen *S. canis *, which further strengthens the case for considering *S. canis* a multi-host pathogen with zoonotic potential.

## Supplementary Data

Supplementary material 1

Supplementary material 2

Supplementary material 3

Supplementary material 4

Supplementary material 5
