# Supplementary material for: Streptococcus canis genomic epidemiology reveals the potential for zoonotic transfer
Source: Microb Genom. 2023 Mar 31;9(3):mgen000974. doi: 10.1099/mgen.0.000974 (PMC10132062; doi:10.1099/mgen.0.000974)
Supplement: Supplementary material 1 [file mgen-9-974-s001.pdf]

Streptococcus canis genomic epidemiology reveals the potential for zoonotic transfer

Davide Pagnossin, William Weir, Andrew Smith, Manuel Fuentes, Juliana Coelho, Katarina Oravcova

SUPPLEMENTARY FIGURES

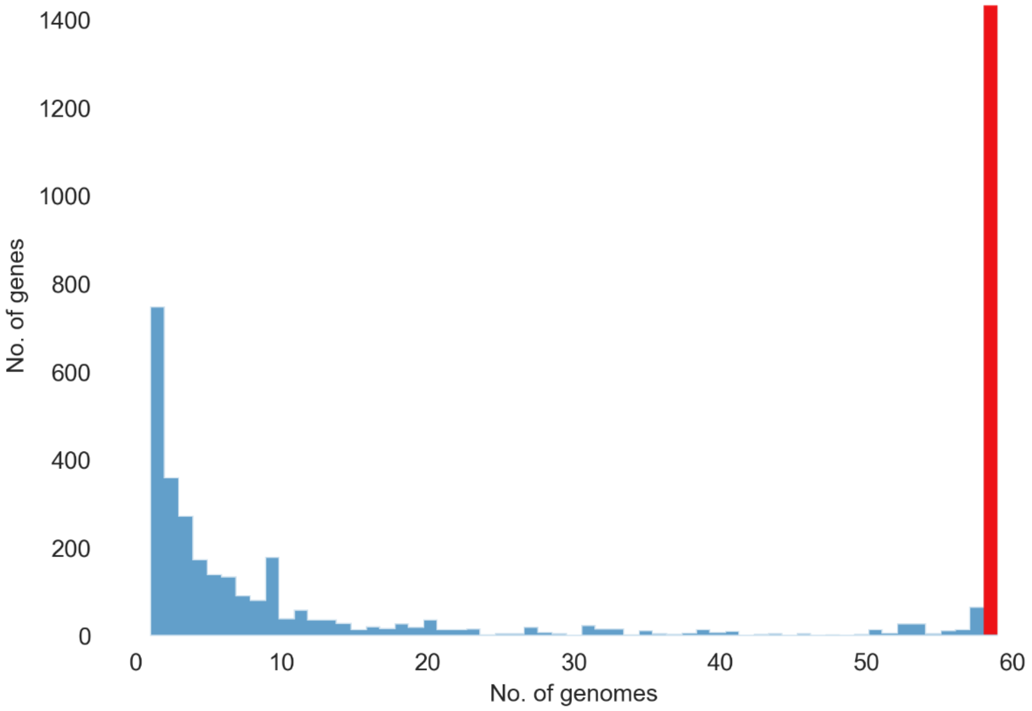

Figure S1. Distribution of the 4,426 *Streptococcus canis* genes detected among the 59 whole genome sequences included in this study. Core genes are colored in red.

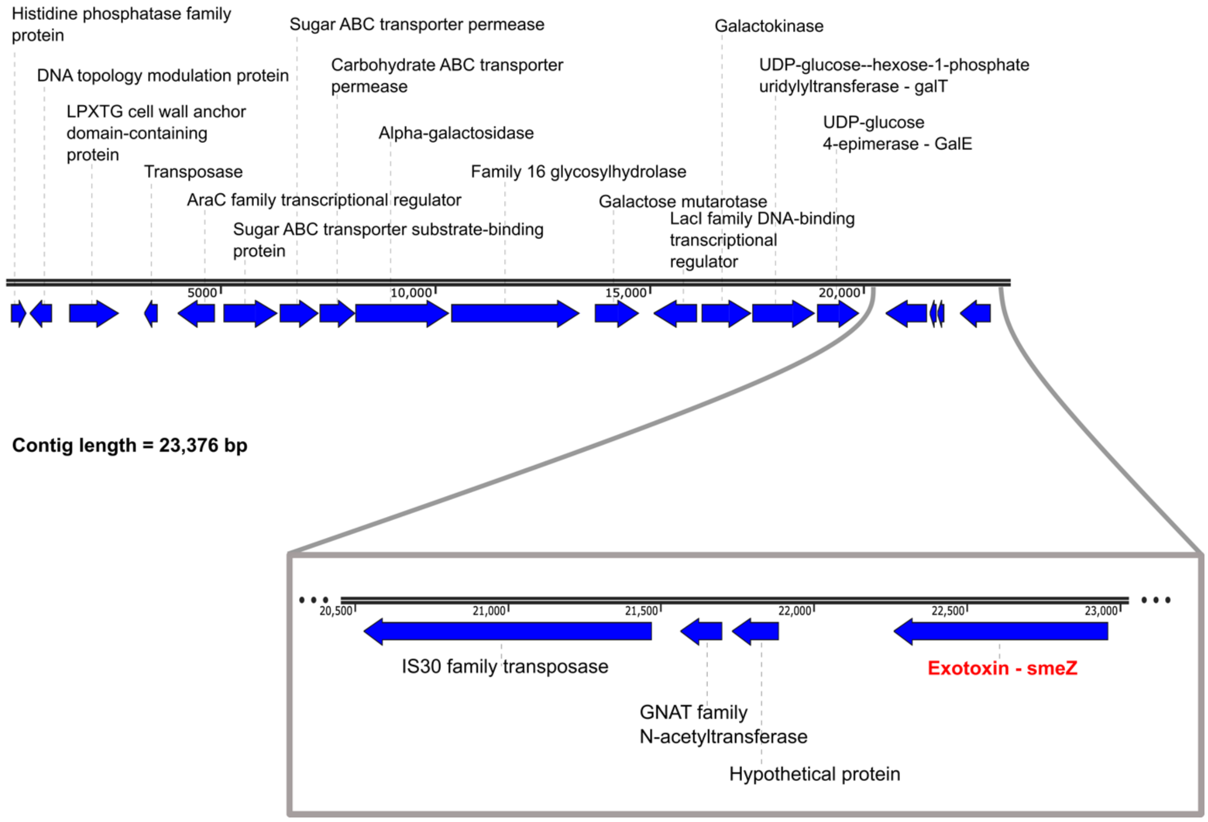

Figure S2. Visual representation of the contig containing the *smeZ* gene in the *Streptococcus canis* D.FMV2238 genome. Genes are annotated according to their function and/or product. Annotations were obtained from publicly available records (accession number: 2238-02).

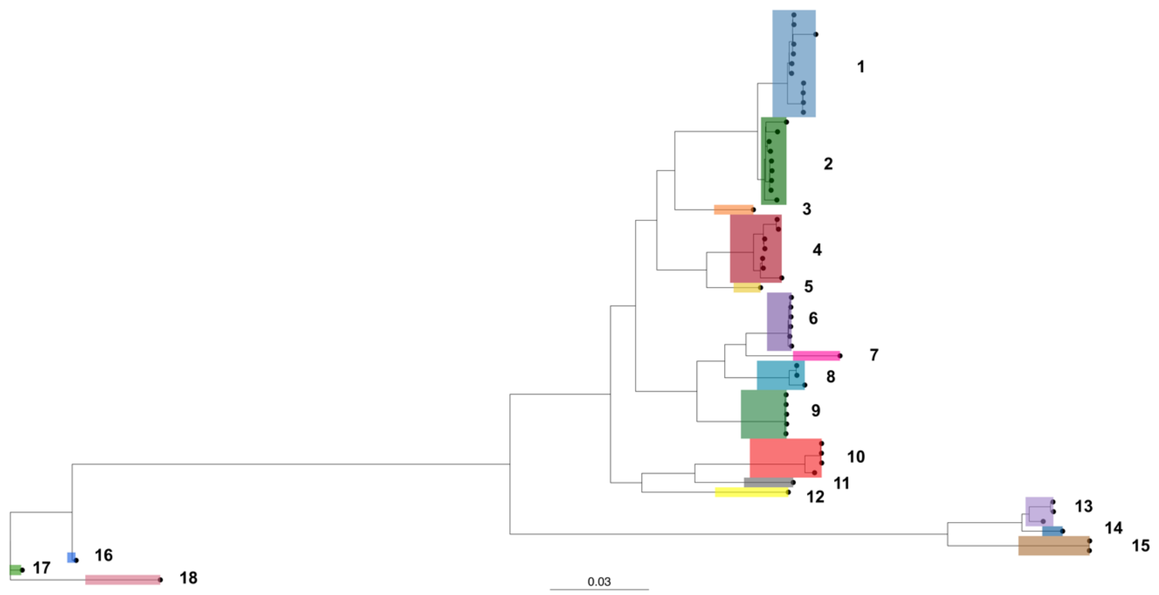

CGS cluster	Within-cluster pairwise SNP distance
1	Min:22<br>Max:1586<br>Avg:606
2	Min:120<br>Max:1238<br>Avg:579
4	Min:61<br>Max:1904<br>Avg:896
6	Min:73<br>Max:225<br>Avg:154
8	Min:47<br>Max:847<br>Avg:578
9	Min:57<br>Max:97<br>Avg:83
10	Min:69<br>Max:958<br>Avg:514
13	Min:196<br>Max:1368<br>Avg:972
15	Min:20<br>Max:20<br>Avg:20

CGS cluster	Between-cluster pairwise SNP distance
1	
	1624
2	
	5769
3	
	5924
4	
	3741
5	
	7690
6	
	4713
7	
	6059
8	
	6135
9	
	10791
10	
	7033
11	
	8786
12	
	21543
13	
	2191
14	
	8505
15	
	25676
16	
	2749
17	
	5470
18	

Pairwise SNP distance - whole tree

Min:20
Max:26610
Avg:10676

Figure S3. *Streptococcus canis* core SNP phylogeny displaying the eighteen core genome SNP (CGS) clusters identified with TreeCluster using a threshold value of 0.017. For clusters composed of more than one isolate, a summary of the

within-cluster pairwise SNP distance is provided. The pairwise SNP distance between the isolates closest to the tree root within each of two adjacent clusters is also reported as a measure of between-cluster distance.

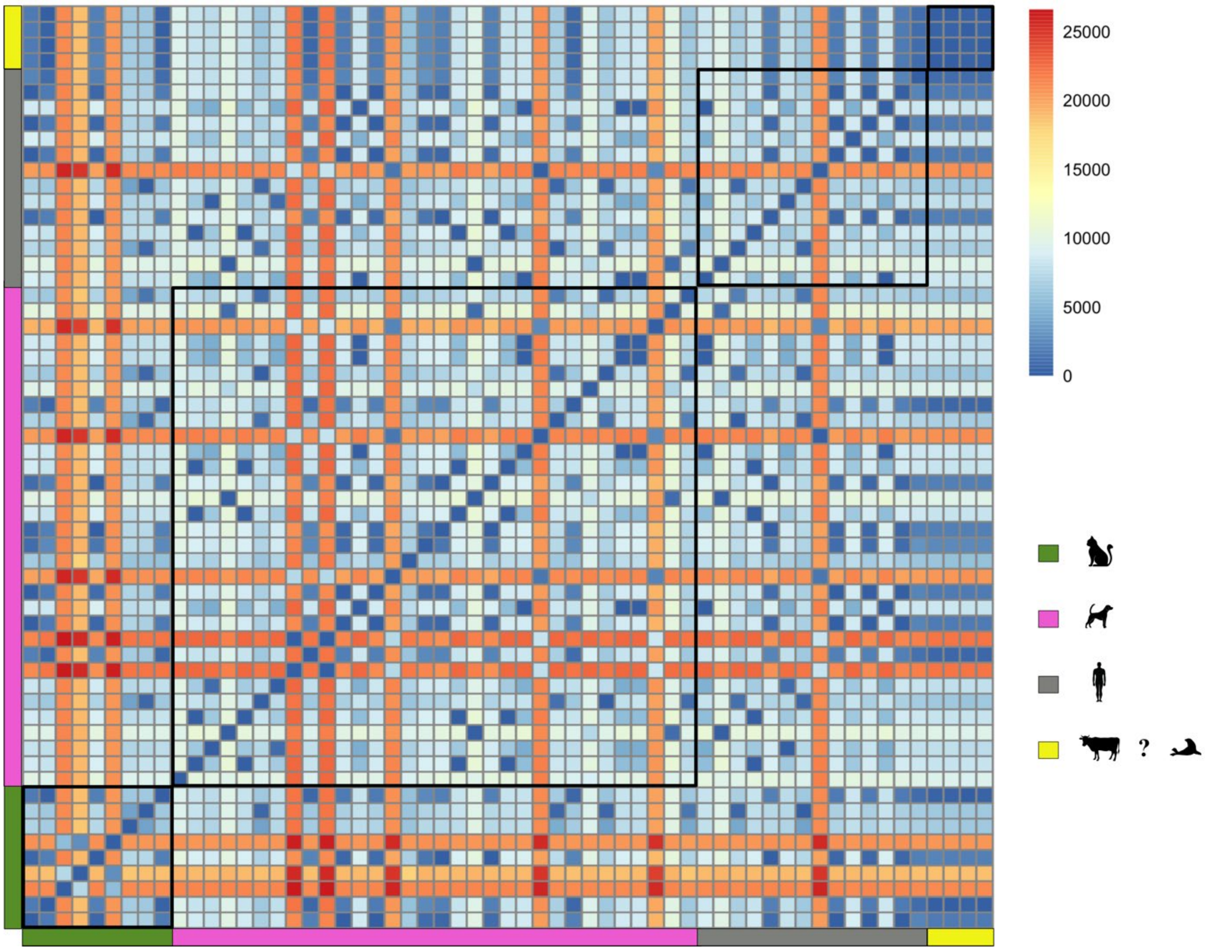

Figure S4. Pairwise core SNP distances of the 59 whole genome sequences analysed, arranged according to the host from which they were isolated. Black squares highlight pairwise distances of isolates from the same hosts or category of hosts.
